# Pediatric vision screening using the plusoptiX A12C photoscreener in Chinese preschool children aged 3 to 4 years

**DOI:** 10.1038/s41598-017-02246-6

**Published:** 2017-05-17

**Authors:** Dan Huang, Xuejuan Chen, Xiaohan Zhang, Yue Wang, Hui Zhu, Hui Ding, Jing Bai, Ji Chen, Zhujun Fu, Zijin Wang, Hu Liu

**Affiliations:** 1 Department of Ophthalmology, The First Affiliated Hospital with Nanjing Medical University, Nanjing, China; 2 Maternal and Child Healthcare Hospital of Yuhuatai District, Nanjing, China; 3 Nanjing Children’s Hospital, Nanjing, China

## Abstract

This study evaluated the performance of plusoptiX A12C in detecting amblyopia risk factors (ARFs) in Chinese children aged 3-to-4-year. PlusoptiX examination was successfully conducted among 1,766 subjects without cycloplegia to detect refractive error, asymmetry and media opacity. Cycloplegic retinoscopy (CR) was conducted on 357 children suspected of having vision abnormalities. Statistical differences between CR and the device were confirmed using the mean spherical value (+1.41 ± 0.87 D versus +1.14 ± 0.81 D), cylindrical value (−0.47 ± 0.64 versus −0.84 ± 0.78) and spherical equivalent (SE) value (+1.17 ± 0.84 D versus +0.72 ± 0.64 D) (all *P* < 0.0001). In the emmetropia group, the differences were statistically significant for the cylinder and SE (all *P* < 0.0001) but not the sphere (*P* = 0.33). In the hyperopia group, the differences were statistically significant for the sphere, cylinder and SE (all *P* < 0.0001). For refractive and strabismic ARFs detection, the sensitivity, specificity, positive predictive value, and negative predictive value were calculated, respectively.

## Introduction

Approximately 1% to 3% of preschool-aged children have amblyopia^1^, a neurological vision disorder attributed to abnormal binocular interaction or visual deprivation during early life. Amblyopia causes vision loss and impaired binocular function in both childhood and adult populations. Although conducting vision screening in school children more than 6 years old is easier, evidence suggests that younger children are more responsive to amblyopia treatment than children older than 7^2, 3^. However, conducting successful visual acuity test in children by using a vision chart is challenging and highly dependent on the cooperation of the children and the experience of the screener.

Instrument-based screening is quick and requires minimal cooperation of the child; therefore, children younger than 4 years old can benefit from this method^4^. The plusoptiX photoscreener, a newly designed screening tool, can assess both eyes simultaneously and is approved by the US Food and Drug Administration. This device uses infrared images of the eye’s red reflex to estimate refractive error, media opacity, ocular alignment and other factors, such as ptosis, all of which increases the risk of a child from developing amblyopia.

Many studies have investigated the performance of the plusoptiX photoscreener for detecting amblyopia risk factors (ARFs)^5^. Given the update of the guidelines of the American Association for Pediatric Ophthalmology and Strabismus (AAPOS) for automated preschool vision screening in 2013, evaluating this device according to the new criteria is necessary^6^. However, the plusoptiX photoscreener has not been applied widely in China, and the only device reporting on the performance of this device in Chinese children utilized the AAPOS 2003 guidelines^7^. Meanwhile, the sixth generation of the devices, including S12 and A12, have not been evaluated fully.

In the present study, we evaluate the performance of the plusoptiX A12C in detecting ARFs in Chinese children aged 3 to 4 years on the basis of the 2013 AAPOS guidelines. This analysis is part of the ongoing prospective Yuhuatai Pediatric Eye Disease Study (YPEDS).

## Results

### Characteristics of study population

A total of 1,818 children aged 3 to 4 years (mean age ± SD: 40.85 ± 3.43 months) agreed to undergo comprehensive eye examinations. Among the subjects, the number of boys (969, 53.3%) is slightly greater than the girls (849, 46.7%). In the 1,818 children, 11 uncooperative children were not tested by the plusoptiX (testability: 99.39%). Among the remaining 1,807 children, the plusoptiX A12C failed to test 41 children (Table 1).

**Table 1:** Characteristics of Study Population.

	Number	%
Total	1818	100.00
Gender		
Male	969	53.30
Female	849	46.70
Testability		
Testable	1807	99.39
Success	1766	97.14
Fail	41	2.26
Untestable	11	0.61

### Comparison between the plusoptiX and cycloplegic retinoscopy

Cycloplegic retinoscopy (CR) was available in 357 (20.24%) of the 1,764 children. The plusoptiX and CR were then compared (Table 2). Statistical differences between CR and plusoptiX were confirmed in the mean spherical value (+1.41 ± 0.87 D versus +1.14 ± 0.81 D; average difference, 0.27 D; *P* < 0.0001), mean cylindrical value (−0.47 ± 0.64 versus −0.84 ± 0.78; average difference, 0.37 D; *P* < 0.0001) and mean SE value (+1.17 ± 0.84 D versus +0.72 ± 0.64 D; average difference, 0.46 D; *P* < 0.0001).

**Table 2:** Comparison between the plusoptiX and Cycloplegic Retinoscopy.

	Sphere (D)				Cylinder (D)				SE (D)			
	Mean	SD	95% CI	P value*	Mean	SD	95% CI	P value*	Mean	SD	95% CI	P value*
Total (N = 357)												
Cycloplegic Retinoscopy	1.41	0.87	1.32–1.50	N/A	−0.47	0.64	−0.54–−0.41	N/A	1.17	0.84	1.08–1.26	N/A
The plusoptiX	1.14	0.81	1.06–1.22	N/A	−0.84	0.78	−0.92–−0.76	N/A	0.72	0.64	0.65–0.78	N/A
Difference*	0.27	0.95	0.17–0.37	<0.0001	0.37	0.56	0.31–0.43	<0.0001	0.45	0.86	0.37–0.54	<0.001
Myopia (N = 3)												
Cycloplegic Retinoscopy	−0.42	1.13	−3.21–2.38	N/A	−1.50	1.80	−5.98–2.98	N/A	−1.17	0.29	−1.88–−0.45	N/A
The plusoptiX	−0.33	1.61	−4.33–3.66	N/A	−1.67	1.15	−4.54–1.20	N/A	−1.17	1.04	−3.75–1.42	N/A
Difference*	−0.08	0.63	−1.64–−1.48	0.84	0.17	0.76	−1.73–2.06	0.74	0.00	0.87	−2.15–2.15	1.00
Emmetropia (N = 298)												
Cycloplegic Retinoscopy	1.17	0.59	1.10–1.24	N/A	−0.47	0.64	−0.54–−0.40	N/A	0.94	0.54	0.88–1.00	N/A
The plusoptiX	1.13	0.78	1.04–1.22	N/A	−0.86	0.80	−0.95–−0.77	N/A	0.70	0.59	0.63–0.76	N/A
Difference*	0.05	0.81	−0.05–0.14	0.33	0.39	0.56	0.33–0.45	<0.0001	0.24	0.72	0.16–0.32	<0.0001
Hyperopia (N = 56)												
Cycloplegic Retinoscopy	2.77	0.80	2.55–2.98	N/A	−0.44	0.55	−0.59–−0.30	N/A	2.55	0.69	2.36–2.73	N/A
The plusoptiX	1.29	0.82	1.07–1.51	N/A	−0.72	0.62	−0.89–−0.56	N/A	0.93	0.69	0.75–1.11	N/A
Difference*	1.48	0.68	1.30–1.66	<0.0001	0.28	0.58	0.13–0.44	<0.05	1.62	0.55	1.47–1.77	<0.0001

SE, spherical equivalent; *Comparison between the Plusoptix A12C and cycloplegic retinoscopy; N/A, not applicable.

**Figure 1:**
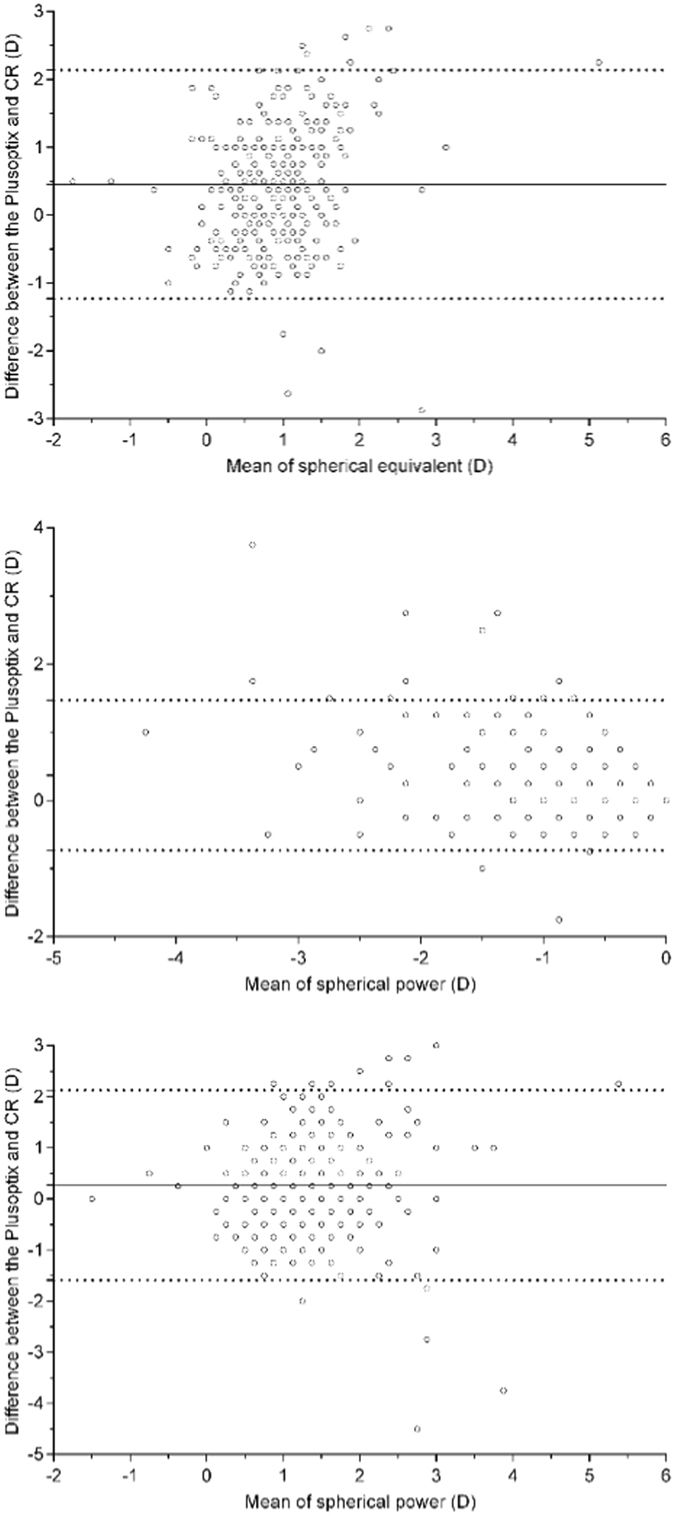
Bland-Altman plots showing the agreement between the plusoptiX A12C and cycloplegia retinoscopy. (**A**) The difference of sphere. (**B**) The difference of cylinder. (**C**) The difference of spherical equivalent.

Figure 1 shows the agreement between the measurements. The 95% limit of agreement (LOA) (CR_Sphere_ − P_sphere_) of the mean spherical value ranged from −1.59 D to +2.13 D in 277 cases with ±1.00 D (77.59%); the 95% LOA (CR_cylinder_ − P_cylinder_) of the cylindrical value ranged from −0.73 D to +1.47 D in 331 cases with ±1.00 D (92.72%); the 95% LOA (CR_SE_ − P_SE_) of the SE value ranged from −1.24 D to +2.16 D in 270 cases with ±1.00 D (75.63%).

According to the SE results, the 357 children were classified into 3 subgroups. In the myopia group of 3 children, the differences were not statistically significant for the sphere (−0.42 ± 1.13 D versus −0.33 ± 1.61 D; average difference, 0.08 D; *P* = 0.84), cylinder (−1.50 ± 1.80 D versus −1.67 ± 1.15 D; average difference, 0.17 D; *P* = 0.74) and SE (−1.17 ± 0.29 D versus −1.17 ± 1.04 D; average difference, 0.00 D; *P* = 1.00). In the emmetropia group of 298 children, the differences were statistically significant for the cylinder (−0.47 ± 0.64 D versus −0.86 ± 0.80 D; average difference, 0.39 D; *P* < 0.0001) and SE (0.94 ± 0.54 D versus −0.70 ± 0.59 D; average difference, 0.24 D; *P* < 0.0001) but not the sphere (1.17 ± 0.59 D versus 1.13 ± 0.78 D; average difference, 0.05 D; *P* = 0.33). In the hyperopia group of 56 children, the differences were statistically significant for the sphere (2.77 ± 0.80 D versus 1.29 ± 0.82 D; average difference, 1.48 D; *P* < 0.0001), cylinder (−0.44 ± 0.55 D versus −0.72 ± 0.62 D; average difference, 0.28 D; *P* < 0.05) and SE (2.55 ± 0.69 D versus 0.93 ± 0.69 D; average difference, 1.62 D; *P* < 0.0001).

### Detection of refractive ARFs

Among the 1,766 tested children, 359 whose refractive measurement values were available or out of range of the device’s setting were included in the analysis of the accuracy of detecting refractive ARFs.

Only 14 children were confirmed to have refractive ARFs according to the criteria of AAPOS (3.90%), including those without myopia, 2 with hyperopia, 10 with astigmatism and 3 with anisometropia. One child was simultaneously diagnosed with hyperopia and anisometropia. The sensitivity and specificity values are displayed in Table 3. The sensitivity ranged from 92.86% to 100%, specificity ranged from 49.57% to 94.49%, positive predictive value (PPV) ranged from 7.45% to 40.63% and negative predictive value (NPV) ranged from 99.69% to 100%, according to the different referral criteria recommended by the manufacturer (Table 3)^6, 8^. The analysis of the adjusted optimal cutoff for detecting refractive ARFs was not conducted because of the small sample size of children with refractive ARFs.

**Table 3:** Accuracy for detecting refractive amblyopia risk factors.

	Age (month)	Myopia (D)	Hyper (D)	Astig (D)	Aniso (D)	Sensitivity (%)	Specificity (%)	PPV (%)	NPV (%)
Manufacturer (sensitivity)	30–50	≤−1.00	≥+1.00	≥1.00	≥1.00	100.00	49.57	7.45	100.00
Matta/Silbert	36–72	≤−1.00	≥+1.25	≥1.00	≥1.25	100.00	57.68	8.75	100.00
AAPOS 2013	31–48	<−3.00	>+4.00	>2.00	>2.00	92.86	94.49	40.63	99.69
ABCD 2012	8–72	≤−2.25	≥+2.50	≥2.25	≥1.00	100.00	90.72	30.43	100.00
Manufacturer (specificity)	36–72	≤−1.50	≥+2.50	≥1.50	≥1.00	100.00	82.03	18.42	100.00

PPV, positive predictive value; NPV, negative predictive value.

### Detection of strabismic ARFs

In addition to the 1,766 successfully tested children, 13 children had refractions that were not measured but had asymmetry measurements that were included in the analysis for the detection of strabismic ARFs. In the 1,779 children, 48 children were diagnosed with strabismus, including 44 with intermittent exotropia, 1 with constant exotropia and 3 with esotropia. However, only 1 child with accommodative esotropia was referred by the device on the basis of the ≥10° criteria for asymmetry (sensitivity 25.00%, specificity 99.83%, PPV 25.00%, NPV 99.83%). Three other children with intermittent exotropia met the ≥5° criteria of asymmetry recommended by the manufacturer (sensitivity 100.00%, specificity 96.11%, PPV 5.48%, NPV 100.00%) (Table 4).

**Table 4:** Accuracy for detecting strabismic amblyopia risk factors.

Criteria	Sensitivity (%)	Specificity (%)	PPV (%)	NPV (%)
Asymmetry ≥5°	100.00	96.11	5.48	100.00
Asymmetry ≥10°	25.00	99.83	25.00	99.83

PPV, positive predictive value; NPV, negativepredictive value.

### Children testing failing

Table 5 provides the detailed diagnosis of the 41 children who failed the test and 11 untested children. Among these children, 20 have vision abnormalities, including 12 with amblyopia, 2 with myopia, 7 with hyperopia, 7 with astigmatism, 6 with anisometropia, 4 with strabismus and 2 with congenital persistent pupillary membrane. The other 28 children were identified to have normal vision.

**Table 5:** Diagnosis of children who failed to be tested or were not tested.

Diagnosis	Number	%
Abnormal	20	38.46
Amblyopia	12	23.08
Myopia <−3.0 D	2	3.85
Hyperopia >4.0 D	7	13.46
Astigmatism >2.0 D	7	13.46
Anisometropia >2.0 D	6	11.54
Persistent pupilary membrane	2	3.85
Strabismus	4	7.69
Normal	32	61.54
Total	52	100.00

## Discussion

The present study evaluated the performance of the plusoptiX A12C in detecting ARFs in preschool Chinese children. As the sixth generation product, the plusoptiX A12C is a portable instrument with rechargeable batteries and requires half the time of the VA-based vision screening. Thus, the plusoptiX A12C can be applied not only in clinical settings but also in community settings^9^. This device uses a smiling face with flashing lights as the fixation target and has a warble sound to catch the attention of young children. The average number of screenings to obtain a reliable result was less than three in 3-year-old children^10^. In this study, almost all children were testable (testability: 99.39%); this finding was consistent with previous reports^11, 12^.

The plusoptiX has been reported by the majority of studies to underestimate children’s refractive error and to have higher accuracy in myopic children than hyperopic children^13–15^. In the present study, the plusoptiX A12C led to a considerable shift towards myopic values (0.45 D), particularly in the hyperopia group (1.62 D), because of normal accommodation. The obvious disparity should be counteracted by appropriate cut-off value. During further analysis, the spherical values agreed well with the emmetropia group (*P* = 0.33) compared with the hyperopia group (*P* < 0.0001). Regarding the cylindrical results, several researchers agreed with the consistency of the cylindrical power between the device and CR^13, 15^, whereas others did not^16, 17^. In the present study, the plusoptiX overestimated the the cylindrical power in all groups, thus resulting in the partial or total underestimation of SE (all *P* < 0.0001). The consistency of the results in the myopia group was not analysed because of the small sample size of three children in these primarily children.

Given the high prevalence of ARFs at 15% to 20%^18, 19^, the majority of children with ARFs do not develop amblyopia, as confirmed by a longitudinal follow-up study^20^. Children with deep amblyopia are less likely to improve by spectacle treatment alone, thus these children must be identified at a younger age^21^. Results suggest that preschool vision screening devices should aim to detect only the greatest magnitude of anisometropia at younger ages by focusing on high specificity and low sensitivity. Reevaluating the majority of studies on the plusoptiX according to the new guidelines updated in 2013 is necessary to reduce the referral rate for young children by raising the threshold referral values.

Previous studies have reported that the plusoptiX has low sensitivity for detecting strabismus, particularly in strabismus with small angle^12, 22^. In the present study, the majority of the strabismic children have controllable intermittent exotropia (44/48, 91.67%). In the other 4 children, only 1 child was referred by the device under the criteria as asymmetry ≥10° (sensitivity: 25.00%).

The plusoptiX has been demonstrated to be a useful screening tool compared with other devices, including the Suresight, SPOT, Retinomax and MTI^23–26^. Several studies have investigated the accuracy of the plusoptiX in detecting ARFs in the pediatric population by modifying the referral criteria to improve the clinical utility of the device^5, 27, 28^. The results of these studies suggested that the referral criteria should be chosen according to local conditions and interval of vision screening^5, 29^. A criteria with higher sensitivity may be more suitable for children with poor access to vision care and vice versa. The sensitivity and specificity of detecting refractive ARFs according to the five criteria recommended by the manufacturer are displayed in Table 3, which shows that the sensitivity ranged from 92.86% to 100%, while the specificity of the criteria of the manufacturer (sensitivity) (49.57%) and Matta/Silbert (57.68%) were lower with the lowest threshold and the criteria of AAPOS 2013 and ABCD 2012 had the highest specificity (AAPOS 2013: 94.49%; ABCD 2012: 90.72%) and PPV (AAPOS 2013: 40.63%; ABCD 2012: 30.43%). In this population-based study, only 14 successfully tested children were confirmed to have refractive ARFs thus increasing the error in analyzing the sensitivity and specificity of the refractive ARFs. By using the AAPOS guidelines updated in 2013, a study on 3-year-old children reported a similar lower PPV of 51% than other studies in representative preschool populations^10^. Such observations can be explained by the more stringent criteria and earlier age range of these two studies.

Approximately half of the 52 children (2.86%) who were not tested successfully were diagnosed with ARFs, thus indicating that such children with high risk for amblyopia should be referred. The small height of palpebral fissure and the occlusion of eyelashes may explain the failure of assessment in the other half of the children.

The advantages of this study include its population-based design (for strabismic ARFs), one of the largest sample size of preschool children, a specific age norm, the newest device and the adopted guidelines of AAPOS updated in 2013. Additional information was provided regarding the strabismic ARFs and children who were not tested. However, this study has several limitations. Although guaranteed by comprehensive eye examinations including refractive status by table-mounted autorefractor and CR, cycloplegia examination was not performed in all included children, thus causing deviation in the analysis, resulting that the detection of refractive ARFs was actually in clinic settings. Only strabismic ARFs was detecting in screening settings. Furthermore, due to the low incidence of refractive error and strabismus in a healthy population, the number of children identified with ARFs was too small to evaluate the accuracy of this device and adjust the optimal cut-off for detecting both refractive and strabismic ARFs. Moreover, the subgroups by refractive state suffers from very unequal sample sizes limited by the specific age norm.

In conclusion, this investigation has shown the statistically significant difference in SE values between the plusoptiX A12C and CR in Chinese children aged 3 to 4 years. We found that the device was accurate in evaluating the spherical value of children with emmetropia. Children who failed the test or were untested should be considered to be at high risk for amblyopia. By using the appropriate criteria, the plusoptiX A12C can be a useful device for detecting refractive ARFs but not strabismic ARFs in preschool children aged 3 to 4 years.

## Methods

### Study design and population

The YPEDS is a population-based vision screening study that aims to establish a systematic database on refraction, visual acuity, ocular biometric parameters, ocular position and other ophthalmic measures. This study also aims to explore the rule for vision development and estimate the occurrence of common pediatric ocular disorders in preschool children aged 3 to 6 years in the Yuhuatai District, Nanjing, China. The YPEDS used inclusion and exclusion criteria similar to those employed in the MEPEDS^30^. After confirmation from parents or legal guardians that the participants are all residents of Yuhuatai District, all children born between September 2011 and August 2012 and those about to enter kindergarten in Yuhuatai District were invited to participate in the study and to undergo comprehensive health examination and vision examination. All children were 3-to-4-year during the examinations.

This study was approved by the Ethics Committee of Nanjing Medical University and was conducted in accordance to the tenets of the Declaration of Helsinki. Written informed consent was obtained from the parents or legal representatives of all participating children.

### Examination

Comprehensive eye examinations were performed by a team of two optometrists and two ophthalmologists who were trained and certified using standardized study protocols as previously described in the MEPEDS^30^. Basic participant information including name, gender, nation, birth day and examination date, was recorded during the clinical visit. The examinations included anthropometric parameters, distance visual acuity (using HOTV VA chart at a distance of 3 m), anterior segment examination, autorefraction, plusoptix A12C photorefraction (PlusoptiX GmbH, Nuremberg, Germany), cover test at distant and near fixation, ocular motility, fundus examination and ocular biometric parameters.

The plusoptiX A12C was placed at a distance of 1 meter in front of the children under dim ambient light. Examination with the device was conducted simultaneously on both eyes by a trained optometrist in accordance with the manufacturer’s instructions. The refraction setting ranged from −7.00 D to +5.00 D for spherical and cylindrical values, respectively, with increments of 0.25 D. The asymmetry ranged from 0° to 25° with increments of 0.1°. When the SE was out of the range, the measurement value only displayed ‘Hyperopia’ or ‘Myopia’. Until it succeeded, the test were conducted for five times at least.

The refraction status and the BCVA of children who volunteered or with abnormal results in the examinations of the PlusoptiX, the table-mounted autorefraction (R-F10, Cannon, Tokyo, Japan), ocular alignment, ocular movement, pupil distance, distance VA and ocular biometric parameters (IOLMaster, Carl Zeiss Meditec, Jena, Germany) were advised to undergo further evaluation with topical 1.0% cyclopentolate (Cyclogyl, Alcon, Belgium). Two drops were instilled 5 minutes apart, with the third drop administered after 20 minutes. After an additional 15 minutes, cycloplegia was evaluated and considered complete in the absence of light reflex. If light reflex was detected, another drop of cyclopentolate was administered; the light reflex was tested after 15 minutes, and refractive errors were measured by CR.

### Definition

According to the AAPOS guidelines updated in 2013, automated preschool vision screening for ARF should detect refractive ARFs, including astigmatism >2.0 D, hyperopia >4.0 D, anisometropia >2.0 D and myopia <−3.0 D, and nonrefractive ARFs, including media opacities (>1 mm) and manifest strabismus (>8 PD in primary position) in children aged 31 to 48 months^6^. Intermittent exotropia and well-controlled deviations were not considered as strabismic ARFs.

### Data analysis

Statistical analyses were performed using the Statistic Product for Service Solution (SPSS) for Windows V.7.0 software (V.22.0, IBM, China). All probabilities quoted are two-sided and were considered statistically significant at less than 0.05. All confidence intervals (CIs) are 95%. Data from the right eyes were analysed to avoid enantiomorphism bias except for the anisometropia calculation^31^.

Data were calculated using the following equations: SE = sphere + (cylinder/2); spherical anisometropia = |sphere (left) − sphere (right)|; and cylindrical anisometropia = |cylinder (left) − cylinder (right)|. The myopia group was defined as SE myopia when ≤−1.00 D, and the hyperopia group was defined as SE hyperopia at ≥+2.00 D.

Descriptive data were presented as mean, standard deviation and frequency. Paired t-test analysis was performed to assess the difference and quantitative relationship of the results. The Bland-Altman plot was used to document the agreement of the measurements. The sensitivities, specificities, PPVs and NPVs were calculated based on 5 sets of criteria recommended by the manufacturer. The receiver operating characteristic curve was employed to select the best cut-off points related to appropriate sensitivity and specificity.
